# Fossilized leftover falls as sources of palaeoecological data: a ‘pabulite’ comprising a crustacean, a belemnite and a vertebrate from the Early Jurassic Posidonia Shale

**DOI:** 10.1186/s13358-021-00225-z

**Published:** 2021-04-29

**Authors:** Christian Klug, Günter Schweigert, René Hoffmann, Robert Weis, Kenneth De Baets

**Affiliations:** 1 Paläontologisches Institut und Museum, Universität Zürich, Karl-Schmid-Strasse 4, 8006 Zurich, Switzerland; 2 Staatliches Museum für Naturkunde, Rosenstein 1, 70191 Stuttgart, Germany; 3 Institute of Geology, Mineralogy and Geophysics, Ruhr-Universität Bochum, 44801 Bochum, Germany; 4 Section Paléontologie 25, Musée national d’histoire naturelle, rue Münster, 2160 Luxembourg City, Luxembourg; 5 GeoZentrum Nordbayern, Fachgruppe PaläoUmwelt, Friedrich-Alexander-University Erlangen-Nürnberg, Loewenichstr. 28, 91054 Erlangen, Germany

**Keywords:** Cephalopoda, Belemnitida, Toarcian, Anatomy, Palaeoecology, Taphonomy

## Abstract

Especially in Lagerstätten with exceptionally preserved fossils, we can sometimes recognize fossilized remains of meals of animals. We suggest the term leftover fall for the event and the term pabulite for the fossilized meal when it never entered the digestive tract (difference to regurgitalites). Usually, pabulites are incomplete organismal remains and show traces of the predation. Pabulites have a great potential to inform about predation as well as anatomical detail, which is invisible otherwise. Here, we document a pabulite comprising the belemnite *Passaloteuthis laevigata* from the Toarcian of the Holzmaden region. Most of its soft parts are missing while the arm crown is one of the best preserved that is known. Its arms embrace an exuvia of a crustacean. We suggest that the belemnite represents the remnant of the food of a predatory fish such as the shark *Hybodus*.

## Introduction

Direct evidence for predation in the form of associated remains of predator and prey is quite rare in the fossil record of most marine deposits (Boucot, 1990; Boucot & Poinar, 2010; Klompmaker et al., 2019). Nevertheless, fossils with prey in the mouth or appendages (i) oesophagus or stomach contents (ii) cololites (iii) and coprolites (iv) as well as regurgitalites (v) with recognizable prey parts provide valuable information about trophic links within food webs.

Sometimes, voracity can be lethal (i). This is evident, when the prey was too large to be swallowed and parts are still within the buccal cavity (oralite sensu Hunt & Lucas, 2012). There are several examples from bony fish, some are quite spectacular due to the size of the animals or other aspects of the prey (Frey & Tischlinger, 2012; Viohl, 1987, 1990). Similar cases were reported recently from cephalopods (Hart et al., 2020; Jenny et al., 2019; Klaschka, 2018; Klug et al., 2021a, b), where bony fish or other coleoids are preserved within the arm crown of Jurassic coleoids.

Reports on fossil stomach contents (ii) are quite common (gastrolite and oesophagolite sensu Hunt & Lucas, 2012), especially from fish (e.g., Ebert, 2012; Kogan & Licht, 2013; Kogan & Romano, 2016; Přikryl et al., 2012; Thies et al., 2019; Tintori, 2019; Wilby & Martill, 1992; Williams 1990). A case was reported from Kansas where a giant, 4.25-m-long *Xiphactinus* had completely swallowed a *Gillicus* of 1.8 m length (Walker, 1982, 2006). A famous complete specimen of the Jurassic shark *Hybodus hauffianus* is particularly spectacular, because its stomach contains over 100 rostra of the belemnite *Acrocoelites* (Brown, 1900; Doyle & Macdonald, 1993; Hoffmann & Stevens, 2020).

Less spectacular but still remarkable, stomach contents have been reported from several fossil cephalopods (e.g., Keupp et al., 2010, 2016; Klug & Lehmann, 2015; Klug et al., 2019; Hoffmann et al., 2020 Landman & Davis, 1988, 2021; Wippich & Lehmann, 2004).

Cololites (iii) may also contain identifiable remains of prey. Argyriou et al. (2015) described cololites of the Triassic fish *Saurichthys*. Another example was published by Zatoń et al. (2017), who found conodonts in a cololite within a Devonian coelacanth.

Coprolites (iv) recently started to receive more attention, partially because modern non-destructive methods permitted a better examination of their contents (e.g., Hunt et al., 2012; Qvarnström et al., 2017, 2019; Schweigert & Dietl, 2012; Vallon, 2012; Zatoń & Rakocinski, 2014). Of course, they did attract scientific interest already much earlier (e.g., Buckland, 1829; Chin, 2002; Gilmore, 1992; Mehl, 1978). The trace fossil *Lumbricaria* occurs in great numbers in Late Jurassic platy limestones. It was recognized as faecal strings of ammonites often containing numerous remnants of planktic saccocomid crinoids (Knaust & Hoffmann, 2020).

Another possibility to preserve traces of predation are regurgitalites (v). Probably, they were widely overlooked because they superficially might resemble coprolites or random accumulations of animal hard parts (Zatoń & Salamon, 2008). Published examples include regurgitated ammonoid shell shards from the latest Devonian (Klug & Vallon, 2019), pterosaur remains that were brought up by some predator (Schweigert et al., 2001) and Early Jurassic vomit containing three more or less macerated actinopterygians (Thies & Hauff, 2012). With their reviews, Hoffmann et al. (2020), Knaust and Hoffmann (2020), and Knaust (2020) wanted to increase awareness for such fossils (see also Vallon, 2012).

Here, we demonstrate that there is another process that produced fossilized traces of predation, i.e. it belongs to the realm of ichnofossils, namely when a predator drops all or parts of its prey, which then is fossilized. It differs from regurgitalites and other bromalites in the fact that it had never really entered the digestive tract (compare Hunt & Lucas, 2012). We first considered to dub these events ‘food falls’, but this term is preoccupied from neontology (e.g., Higgs et al., 2014; Stockton & DeLaca, 1982). Food falls also include whale falls (e.g., Smith, 1989) and do not imply the cause of death of the animal that becomes a benthic food source (Baco & Smith, 2003; Rouse et al., 2004). In contrast, ‘leftover falls’ are regarded as food remains that have been lost for unknown reason during the predator attack. Both, leftover falls and food falls are known from today’s oceans. Further details are discussed below.

The specimen portrayed here is a belemnite associated with crustacean remains from the German Toarcian. Belemnites played a key role in the marine basins of Europe during the Jurassic (Dera et al., 2016; Neige et al., 2021; Rita et al., 2020). There is a growing body of evidence that belemnoids and their relatives preyed upon fish nearly as long as their mantle (Hart et al., 2020; Jenny et al., 2019; Keupp et al., 2010). It is still a matter of debate whether they were ambush predators or able to chase their prey to some extent (Klug et al., 2016). With their pointed beaks (Klug et al., 2010, 2020; Keupp & Mitta, 2015; Lehmann et al., 2016), they were able to hold, immobilize and cut prey. Recently, it was suggested that they might have been able to break ammonite conchs in order to facilitate withdrawal of the soft parts (Klug et al., 2021b).

As far as we know, the Posidonienschiefer Formation (Posidonia Shale) of the Holzmaden region yields the best specimens of belemnites preserving soft-tissues (Reitner & Urlichs, 1983; Riegraf & Hauff, 1983; Schlegelmilch, 1998; Urlichs et al., 1994;). Like the material described by Klug et al. (2021a), the piece described here was collected from the Posidonia Shale by Dieter Weber. For palaeontology, the Posidonia Shale offered a rare combination of a shallow to moderately deep continental marine basin, in which oxygen became depleted numerous times during the Toarcian (Röhl et al., 2001, 2002). At the same time, primary production was high enough to sustain a food web in the water column comprising numerous species of reptiles, fishes, ammonoids, coleoids, crinoids, bivalves and other animals. The trophic interactions are occasionally documented by stomach contents, coprolites, cololites, etc. (Jenny et al., 2019). By contrast, the sediment body and surface was repeatedly inhabited by, e.g., bivalves, crustaceans, gastropods and echinoids, but often for short intervals because of the oxygen fluctuations (Bottjer et al., 2002; Etter & Tang, 2002; Hauff & Hauff, 1981; Riegraf et al., 1984; Röhl et al., 2001, 2002). Often, the oxygen content was too low to sustain scavengers and, in the absence of strong currents, skeletons had a reasonable likelihood to preserve in articulation and soft parts of embedded carcasses could become fossilized (e.g., Etter & Tang, 2002; Reisdorf et al., 2012).

Here, we describe a belemnite from the Toarcian of southern Germany, which holds crustacean remains in its arm crown. In turn, the belemnite displays damage indicative of predation by a larger animal. We use this case to discuss the events that led to this fossil association, we introduce the technical term ‘pabulite’ for fossilized leftover falls and provide a definition, and we put it into the context of the Middle European marine food webs of the Toarcian.

## Materials and methods

The specimen described here was discovered in 1970 by the fossil collector Dieter Weber (Rechberghausen). GS recognized its importance during a visit of his splendid private collection in 2019 and it was subsequently purchased by the Staatliches Museum für Naturkunde Stuttgart (acronym SMNS). Weber extracted the slab from the Posidonienschiefer Formation exposed in the now abandoned Gonser quarry at Ohmden near Holzmaden. For a generalized section see Riegraf et al. (1984: Fig. 20). The finding level of the specimen is the ʻKoblenzerʼ bed, a bituminous marly claystone, which is Early Toarcian, Tenuicostatum Zone (Semicelatum Subzone) in age (Riegraf et al., 1984). This bed, although bituminous, does not show lamination and possibly represented a rather soupy substrate. The ʻKoblenzerʼ in the vicinity of Holzmaden is especially well-known for its articulated belemnite specimens such as the present one (Reitner & Urlichs, 1983; Riegraf & Hauff, 1983; Schlegelmilch, 1998) besides numerous other coleoids (which belong to the genera *Clarkeiteuthis* and *Chondroteuthis*; Fuchs & Weis, 2008; Fuchs et al., 2013) as well as nicely preserved decapod crustaceans of the genera *Uncina*, *Proeryon*, and *Tonneleryon* (Audo, 2016; Audo et al., 2020; Schweigert et al., 2003). Vertebrate remains comprise fishes, ichthyosaurs and marine crocodiles, but these are less frequently found, because the Koblenzer bed underlies the Fleins bed, for which the Posidonia Shale is quarried, and this bed was therefore rarely exposed (Dieter Weber, pers. comm. 2019). For an expanded, but taxonomically partially outdated faunal list see Riegraf et al. (1984).

## Results

**Fig. 1:**
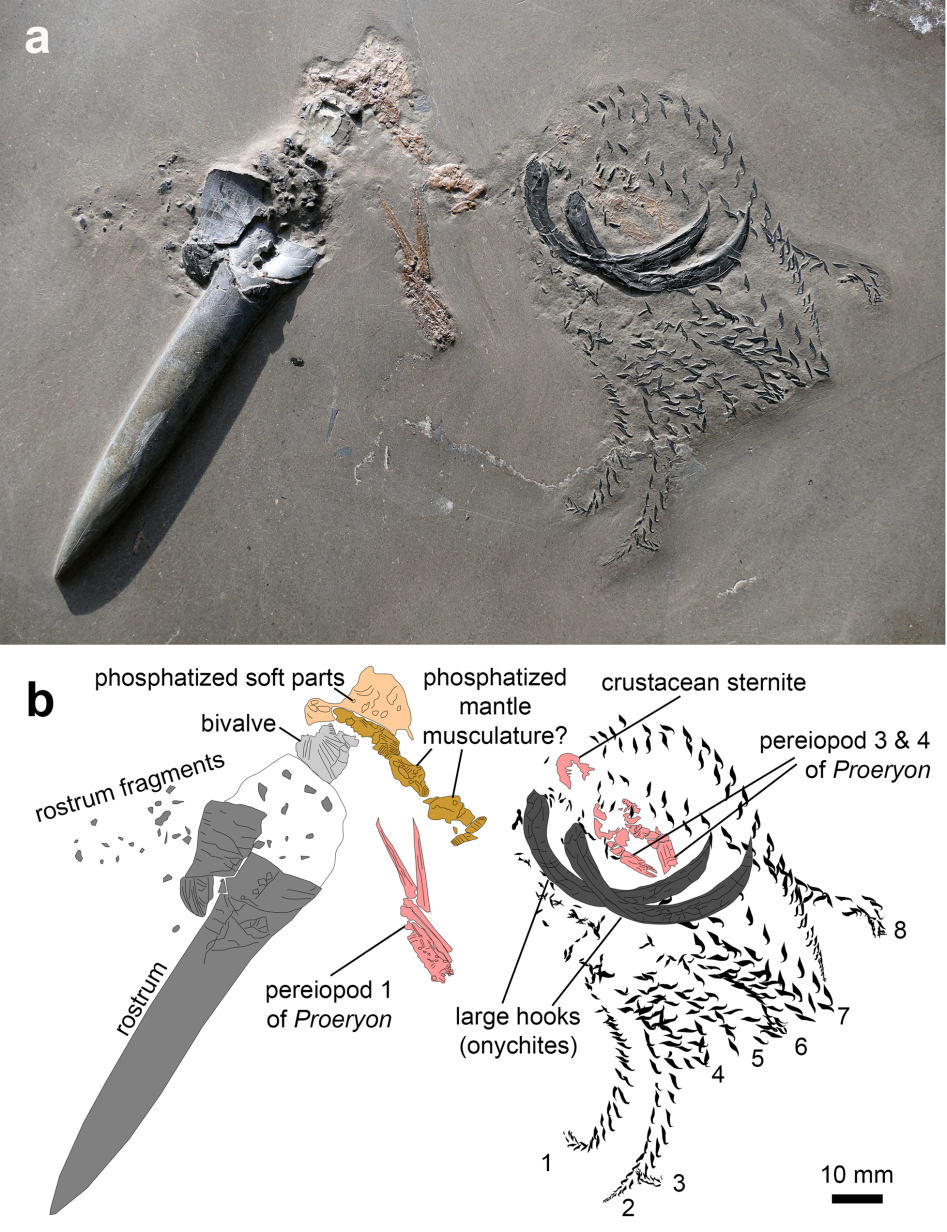
Taphocoenosis of a *Passaloteuthis bisulcata* with preserved arm crown and remains of its prey, SMNS 70514, Early Toarcian, Tenuicostatum Zone, Semicelatum Subzone, Ohmden, Germany. **a** Photo of the specimen and its prey. **b** Camera lucida drawing after **a**

The plate of Posidonia Shale, which we describe here, displays remains of a belemnite and a crustacean as well as a pyritized shell of a pectinid (Fig. 1). The belemnite belongs to the species *Passaloteuthis laevigata* (Zieten, 1831) (for synonymy see Sanders et al., 2015 and Weis et al., 2018), a well-known and frequent belemnite species in the lowermost Toarcian of Europe and Morocco (Doyle, 1990; Riegraf et al., 1984; Sanders et al., 2015; Schlegelmilch, 1998; Weis et al., 2018). It preserves the rostrum, a few soft part remains, and the nearly complete arm crown. The rostrum is nearly 93 mm long including the shards of the fragmented rostrum cavum. The largest shard measures 11 × 13 mm. The rostrum solidum is well preserved and maximally 13 mm wide. The rostrum shows the ventral part exposed. The apex bears fine, short apical striae. The apical part of the rostrum displays an irregular, “pseudo-ventral groove” (Doyle, 1990: p. 21) that does not reach the apex. Such irregular grooves sometimes occur in *Passaloteuthis laevigata* (Schlegelmilch, 1998: p. 51) and are typically pathologically deformed (Doyle, 1990). Around the rostrum cavum, several smaller fragments are scattered. No remains of the phragmocone and proostracum are visible.

**Fig. 2:**
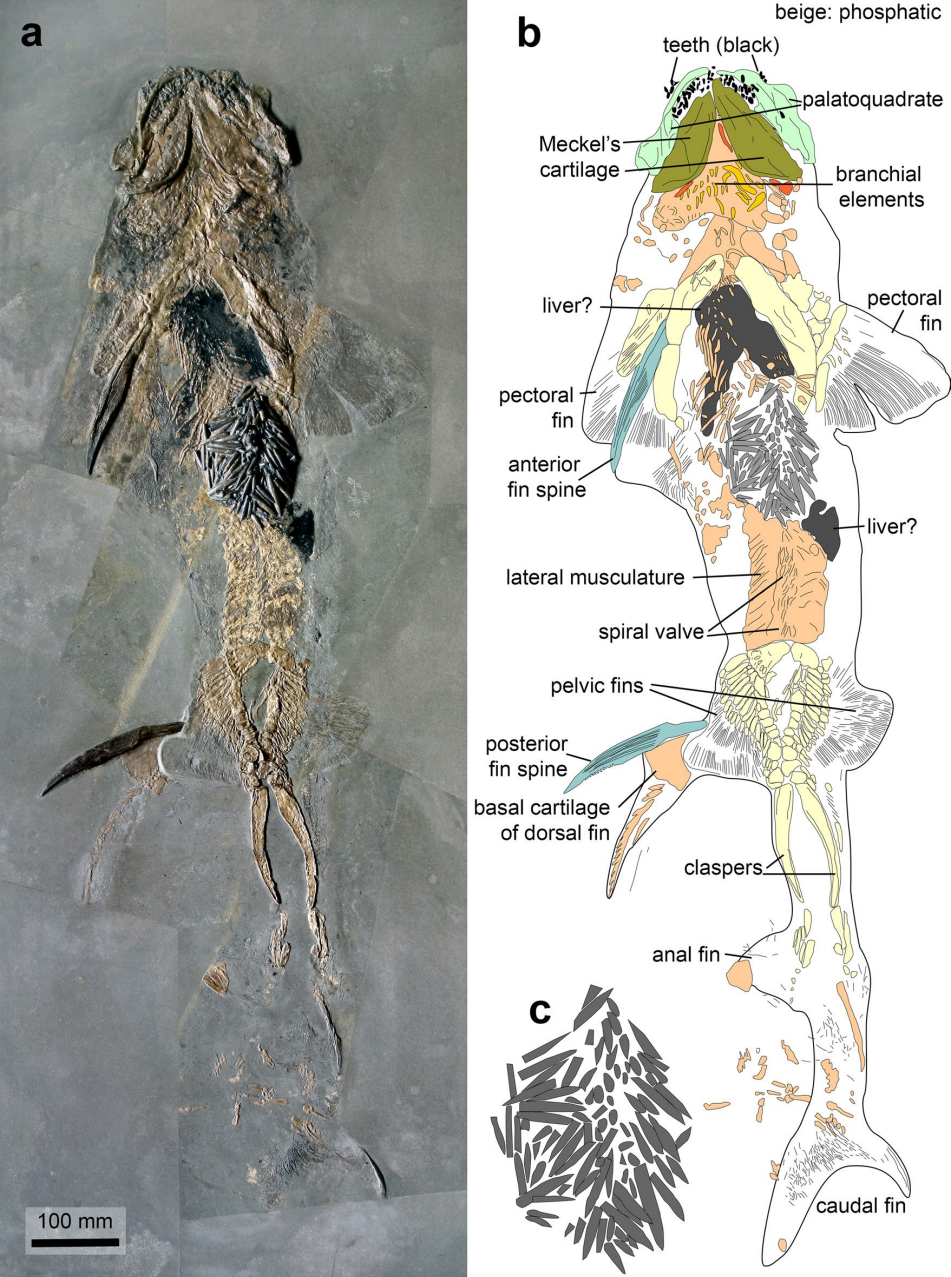
The Early Jurassic elasmobranch *Hybodus hauffianus* with its stomach clogged by belemnite rostra; SMNS 10062, Posidonia Shale, Toarcian, Holzmaden (Germany). **a** Photo taken by R. Böttcher (Stuttgart; copyright Staatliches Museum für Naturkunde in Stuttgart). **b** Camera lucida drawing after a; beige: phosphatic parts; dark grey: parts of the liver(?); middle grey: belemnite rostra in the stomach; yellow: paired fins. **c** Magnified stomach content with 93 visible fragments of rostra (magnified: **b** × 2)

The soft part remains are fragmentary compared to some other specimens (e.g., Schlegelmilch, 1998: pl. A Figs. 1, 2). They can be subdivided into three parts, two of which share a middle brown colour while the third part is of a slightly lighter, beige colour. The colour suggests that these parts are phosphatized. The beige part has an irregular outline and covers a surface of 23 × 10 mm. It shows no distinct patterns. One of the brownish parts is elongate measuring 25 × 5 mm. It shows parallel striations of varying orientation. The other brownish part forms a lump of about 10 mm diameter lacking surface patterns. It is neighboured by two elongate structures of brownish colour. They are striated and together are 10 mm long and 2 mm wide.

All hooks are well preserved and at least four of the arms appear to be complete. The complete arms are labelled 1, 2, 7 and 8 in Fig. 1b. They are between 80 and 90 mm long and display 23 (arm 1), 25 (arm 2), 25 (arm 7), and 28 (arm 8) pairs of micro-hooks. Assuming that this represents the full set of micro-hooks, they would have carried a total of minimum 406 arm hooks including the large pair (see Fuchs & Hoffmann, 2017 for other published specimens with preserved hook pairs). Micro-hook size varies between c. 0.4 mm at the arm tips and 4 mm in the middle. The morphology of micro-hooks for various species has been described elsewhere (Fuchs, 2006; Hoffmann et al., 2017; Reitner & Urlichs, 1983; Riegraf & Hauff, 1983; Schlegelmilch, 1998).

A pair of complete large onychites (mega-hooks following Fuchs & Hoffmann, 2017) is located within the arm crown. ‘*Passaloteuthis paxillosa*’ (= *P. laevigata*) is the only identified belemnite with associated mega-hooks so far (Fuchs & Hoffmann, 2017). Their perfect arrangement between the largely undisturbed arm crown suggests that they were attached to a pair of modified arms very close to the head. They measure 39 mm in their greatest section and are up to 5 mm wide in their slightly compacted state. The proximal ends are rounded, while the proximal ends form sharp tips. They are strongly curved, forming roughly half a circle, which is slightly elliptical with the smaller radius (c. 10 mm) in the proximal part and the larger radius (c. 18 mm) distally. Both display pronounced lateral ridges close to the concave edge. The morphology of the large hooks corresponds well to the parataxon *Onychites uncus* Quenstedt (1856). These mega-hooks were later revised by Engeser (1987).

Several brownish remains of the appendages of a reptantian crustacean are visible in the arm crown and lateral to the place where the proostracum used to be. The latter part is discernible as the claw of a cheliped (pereiopod 1). The complete propodus is 35 mm long and only 6 mm wide. The dactylus is 18 mm long and tapers in height from proximally 3 mm to about 1 mm distally. At the distal tip, the front 1.5 mm are strongly curved. The propodus carries an ornament of fine pustules. The anteroventral part of the propodus shows a saw-like series of pustules. All elements are cracked longitudinally and transversely. The shape of pereiopod 1 is typical of the genus *Proeryon*, which is the most common genus of decapod crustaceans in the Posidonia Shale (Audo et al., 2020).

A cuticular fragment, likely from the same crustacean lies close to the arm bases between the arms and above the bases of the large onychites (Fig. 1). This fragment has a parabolic outline, measures 6 × 6 mm and lacks discernible morphological features. It might belong to the sternum.

The third crustacean remain is surrounded by the large onychites. Two small claws are discernible, suggesting that these are the pereiopods 3 and 4. Only one propodus (probably of pereiopod 4) is completely visible; it is 10 mm long and 2.5 mm wide. The small dactylus is only 2 mm long. The associated proximal parts (probably carpus and merus) measure 11 × 3 mm. Pereiopod 3 is distally covered by one of the large onychites. Its propodus is c. 3.5 mm wide and a bit larger than the other.

## Discussion

### Taphonomical history

Like modern coleoids, Jurassic coleoids were a preferred prey of many vertebrates (Boyle & Rodhouse, 2005a, b; Clarke, 1996; Clarke & Stevens, 1974; Croxall & Prince, 1996; Hess & Toll, 1981; Klages, 1996; Pethybridge et al., 2011; Rodhouse & Nigmatullin, 1996). This has been documented repeatedly by stomach contents (Brinkmann, 2004; Brown, 1900; Doyle & Macdonald, 1993; Hölder, 1955; Hoffmann & Stevens, 2020; Hoffmann et al. 2020; Přikryl et al., 2012; Stewart & Carpenter, 1990; Vullo, 2011; Wilby & Martill, 1992; Williams, 1990). Accordingly, it is not surprising to find traces of such predation in the fossil record. As far as Jurassic belemnites are concerned, there is direct evidence for predation by hybodontid sharks on belemnites: in the Staatliches Museum für Naturkunde in Stuttgart (Germany), a complete specimen of *Hybodus hauffianus* is on display (Fig. 2), which carries a lump of belemnite rostra in its stomach (Doyle & Macdonald, 1993; Hoffmann & Stevens, 2020). This accumulation of hardly digestible rostra was identified as the likely cause of death. The unlucky shark ate too many complete belemnite animals without chewing off the rostra or regurgitating them (93 rostra are visible at the surface, we estimate that there were around 200 in total; Fig. 2c). In most cases, however, belemnites or belemnoteuthid micro-hooks have been reported from large Jurassic marine predators including ichthyosaurs and pachycormid fishes, which might indicate they were specialized in soft-bodied cephalopods or at least used to removing their hard parts (Brinkmann, 2004; Dick et al., 2016; Přikryl et al., 2012; Pollard, 1968; Walker & Brett, 2002; but see Martill, 1986 for a belemnite rostrum in the stomach of metriorhynchid crocodile).

There is also evidence that this was not the normal strategy by Jurassic fish feeding on belemnites. Especially in the platy limestones of the Late Jurassic of the conservation deposit of Nusplingen (Germany), belemnite rostra are often associated with shards of their broken rostrum cavum (Hölder, 1955; Klug et al., 2010; Schweigert, 1999, 2018; Stevens et al., 2014). The arrangement of the fragments evidences that fragmentation did not happen due to compaction but prior to burial. The most plausible interpretation is that vertebrate predators had quickly learned that the rostra are hard, pointed, and difficult to digest and to egest. They thus bit off the soft parts, which were poorly protected by the thin proostracum and dropped fins, posterior mantle and rostrum. We suggest that the association of the complete belemnite arm crown with a complete rostrum and some soft parts represent the remains of the meal of a vertebrate predator, which had learned enough about belemnite anatomy to spare the rostrum. This lends further credibility to the hypothesis that belemnite predation might contribute to belemnite accumulations (so-called battlefields) under certain circumstances (Doyle & Macdonald, 1993), although in deposits lacking soft-tissue preservation likely current concentration and/or condensation are important factors (Rita et al., 2018, 2019; Urlichs, 1971).

Possible culprits of predation on belemnites are the shark *Hybodus hauffianus*, large predatory fish such as representatives of the genera *Pachycormus* (compare Liston et al., 2019) or *Saurorhynchus* as well as of the marine crocodile genus *Steneosaurus* (compare Walker & Brett, 2002). Also adult specimens of ichthyosaurs belonging to the genus *Stenopterygius* have managed to cut out the soft parts with sufficient precision as reflected in their stomach contents containing belemnite mega-hooks (Dick et al., 2016). Pachycormids and marine crocodiles might have been less adapted to this strategy as suggested by the presence of only phragmoteuthid hooks or belemnite hooks associated with a rostrum in their stomach contents, respectively (Martill, 1986; Přikryl et al., 2012). Ichthyosaurs, in addition, become rare towards the Late Jurassic, whereas the characteristically broken rostra of belemnites occur still frequently.

As far as the crustacean is concerned, the incompleteness and the poor sclerotization of the remains of the *Proeryon* suggest that these were part of an exuvia (different example: Klompmaker & Fraaije, 2012). Possibly, the pyritized pectinid shell was originally attached to the cuticle of the crustacean, it might have lain on the sediment already or it could have fallen there accidentally. Exuviae have been widely documented as food items of cephalopods (Boyle & Rodhouse, 2005b; Coelho et al., 1997; Nixon, 1985, 1987, 1988; Rodhouse & Nigmatullin, 1996; Tshudy et al., 1989; Villanueva et al., 2017; Ward & Wicksten, 1980; Ward, 1987; Westermann et al., 2002). Accordingly, we assume that the belemnite was chewing on the exuvia of a *Proeryon* until the belemnite’s demise.

### Leftover falls turn into pabulites

**Fig. 3:**
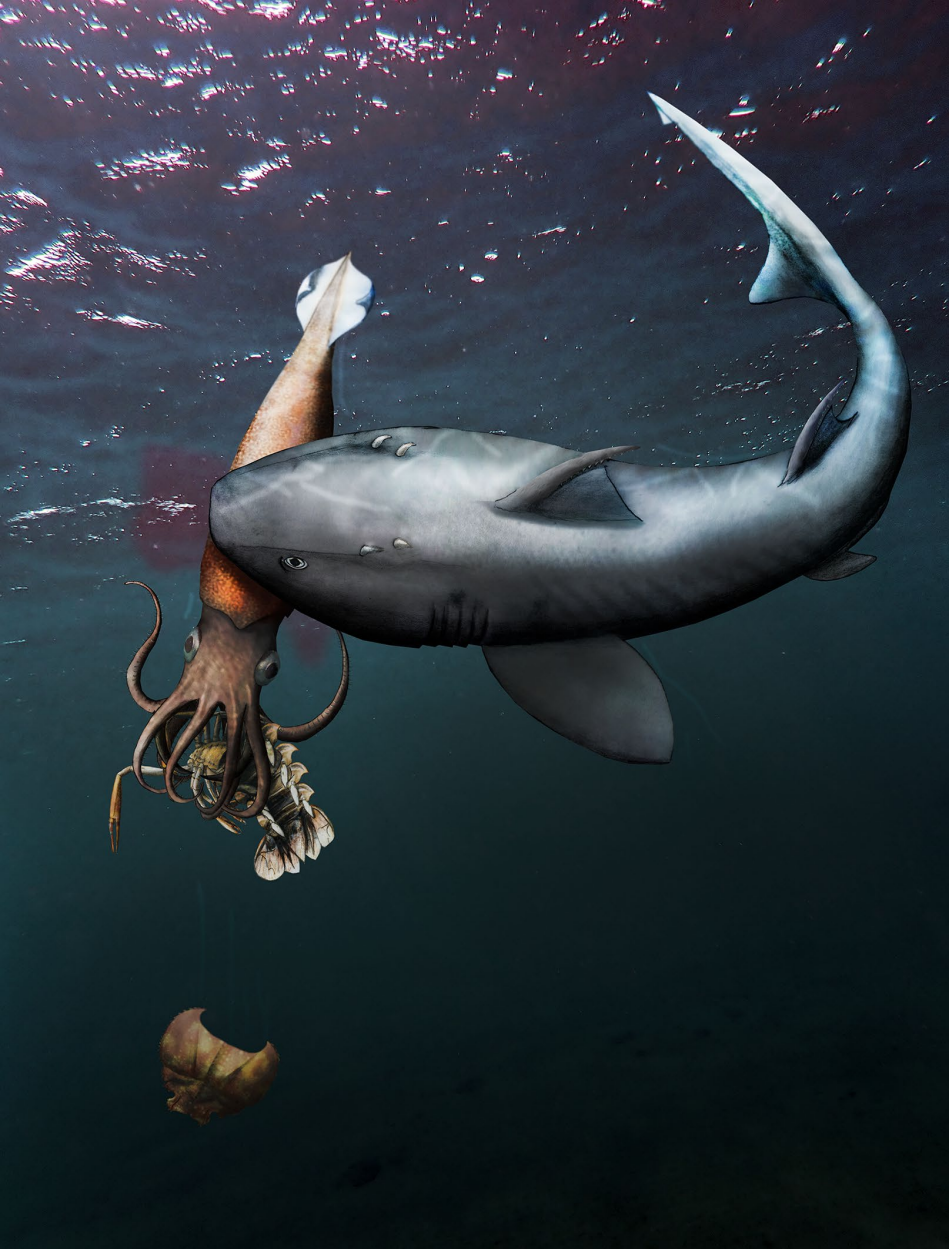
Possible scenario explaining the taphonomy of the belemnite. *Hybodus hauffianus* is known to have fed on belemnites, although it is unclear whether some individuals learned how to avoid the swallowing of the calcitic rostrum. The belemnite *Passaloteuthis laevigata* holds remains of the exuvia of *Proeryon* in its arms

As outlined above, there is good circumstantial evidence that the belemnite described here fell victim to a vertebrate predator (Fig. 3). The predator’s success is supported by the very incomplete preservation of the soft parts. Accordingly, this is herein interpreted as a piece of food, which the predator had dropped. For this kind of fossils, which provide evidence for incomplete predation, we suggest the technical term leftover fall or pabulite. Pabulite is derived from the Latin words *pabulum* for food and the Greek word *lithos* for stone.

Pabulites differ from regurgitalites in the fact that the included parts had never entered the digestive tract beyond the buccal cavity. Accordingly, the diffuse matrix sometimes seen in regurgitalites (Hoffmann et al., 2020; Knaust, 2020; Thies & Hauff, 2012; Vallon, 2012) is missing. Additionally, some of the organs are still in their primary order. It does not really make sense to include pabulites in Digestichnia sensu Vallon (2012) since leftover falls did not even reach the oesophagus and thus have not been digested at all. Having said this, we include pabulites into the group of praedichnia (Ekdale, 1985; Vallon et al., 2016).

Pabulites have been reported repeatedly and are actually quite important in numbers and in palaeontological informative value. For example, many belemnites demonstrably can be classified as pabulites in such cases, where the rostrum cavum is fragmented (Klug et al., 2010; Schweigert, 1999, 2018). Isolated soft parts of an ammonite from the Late Jurassic of Germany described by Klug et al. (2021b) are likely also a pabulite. There are countless examples—few of them published or displayed—of incompletely preserved vertebrate remains from the Late Jurassic Solnhofen-type limestones, such as isolated heads, fins or tails of fishes (e.g., Chellouche, 2016; Dietl & Schweigert, 2011; Maxwell et al., 2021) as well as almost complete but lethally bitten fish, in one case leaving a mortichnium (Schweigert et al., 2016), or even large parts of marine crocodiles (Dietl & Schweigert, 2011; Mäuser, 2015), and abundant crushed ammonite shells (Klug et al., 2021b).

### Large onychites (mega-hooks) and their meaning

Riegraf and Hauff (1983) suggested that the mega-hooks were part of long tentacles with a distal position of the mega-hooks on them. This appears less likely now because in the at least four specimens of soft-tissue belemnites, which preserve the arm crown with both mega-hooks, these large hooks lie close to the mouth and the arm bases, i.e. proximal (e.g., Fuchs & Hoffmann, 2017), which was assumed by Engeser and Clarke (1988). Small onychites appear to be missing entirely in the two modified arms with the mega-hooks.

The presence of very similar forms that may be conspecific (e.g., Reitner & Urlichs, 1983; Riegraf & Hauff, 1983; Schlegelmilch, 1998), which have ten arms with only micro-hooks suggests a sexual dimorphism. Some authors speculate that mega-hooks were only present in males (Engeser, 1987; Stevens, 2010). In the absence of preservation of reproductive organs, however, we cannot sex these specimens. We speculate that the mega-hooks played a role during mating (Engeser & Clarke, 1988; Fuchs, 2006), which would suggest the presence of a hectocotylized arm pair (in recent coleoids, none, one or two arms may be hectocotylized; see the discussion in Klug et al., 2021a). The situation of the crustacean exuvia remains between the mega-hooks suggests that they were also used occasionally to hold on to prey.

## Conclusions

We describe a unique specimen of the Toarcian belemnite *Passaloteuthis laevigata* form the Toarcian Posidonia Shale of Ohmden near Holzmaden (Germany). It is remarkable because of the exceptionally complete in situ preservation of the arm crown with most arm hooks including a complete pair of large onychites. Additionally, the arm crown embraces exuvia remains of the decapod *Proeryon*. Remarkably, most of the belemnite soft parts between the arm crown and the calcitic rostrum are missing. We suggest that this represents remains of a meal of a vertebrate predator, possibly of the Early Jurassic shark *Hybodus hauffianus*. This is remarkable, because it informs about the behaviour of a cephalopod and a vertebrate predator.

Additionally, we use this occasion to introduce the term leftover fall for such remains of a meal and pabulite for the fossilized remains of which. Diverse pabulites have been reported before. They are valuable sources of palaeobiological information and deserve more attention.

## Data Availability

The single specimen illustrated and described is stored at the Staatliches Museum für Naturkunde in Stuttgart, Germany.
